# Supplementary material for: A tough and robust hydrogel constructed through carbon dots induced crystallization domains integrated orientation regulation
Source: Nat Commun. 2025 Jul 5;16:6221. doi: 10.1038/s41467-025-61535-1 (PMC12228686; doi:10.1038/s41467-025-61535-1)
Supplement: Supplementary file 1 — Supplementary Information [file 41467_2025_61535_MOESM1_ESM.pdf]

Supplementary Information

A tough and robust hydrogel constructed through carbon dots induced crystallization domains integrated orientation regulation

Huanxin Huo[1,2,#], Jingjie Shen[1,2,#], Jianyong Wan[1,2,3,*], Haoran Shi[1,2], Hongxing Yang[1,2], Xin Duan[1,2], Yihong Gao[1,2], Yumeng Chen[1,2], Feng Kuang[1,2], Hongshan Li[1,2], Long Yang[1,2,3,*], Guanben Du[1,2,3,*]

[1] Yunnan Province Key Lab of Wood Adhesives and Glued Products, Southwest Forestry University, Kunming 650224, China

[2] College of Materials and Chemical Engineering, Southwest Forestry University, Kunming 650224, China

[3] International Joint Research Center for Biomass Materials, Southwest Forestry University, Kunming 650224, China

[#] These two authors contributed to this work equally

* Corresponding Authors

Corresponding: jywan@swfu.edu.cn (J. Wan); lyang@swfu.edu.cn (L. Yang); guanben@swfu.edu.cn (G. Du)

Supplementary Table 1. The compositions of the PVA, PVA-CA and PVA-CDs hydrogels.

Sample	PVA (g)	CA (g)	CDs (g)	Water (g)
PVA$_{10\%}$	10			90
PVA$_{20\%}$-CA$_{10\%}$(PVA-CA)	20	8		72
PVA$_{10\%}$-CA$_{10\%}$	10		9	81
PVA$_{15\%}$-CDs$_{10\%}$	15		8.5	76.5
PVA$_{20\%}$-CDs$_{10\%}$(PVA-CDs)	20		8	72
PVA$_{25\%}$-CDs$_{10\%}$	25		7.5	67.5
PVA$_{20\%}$-CDs$_{1\%}$	20		0.8	79.2
PVA$_{20\%}$-CDs$_{5\%}$	20		4	76
PVA$_{20\%}$-CDs$_{15\%}$	20		12	68

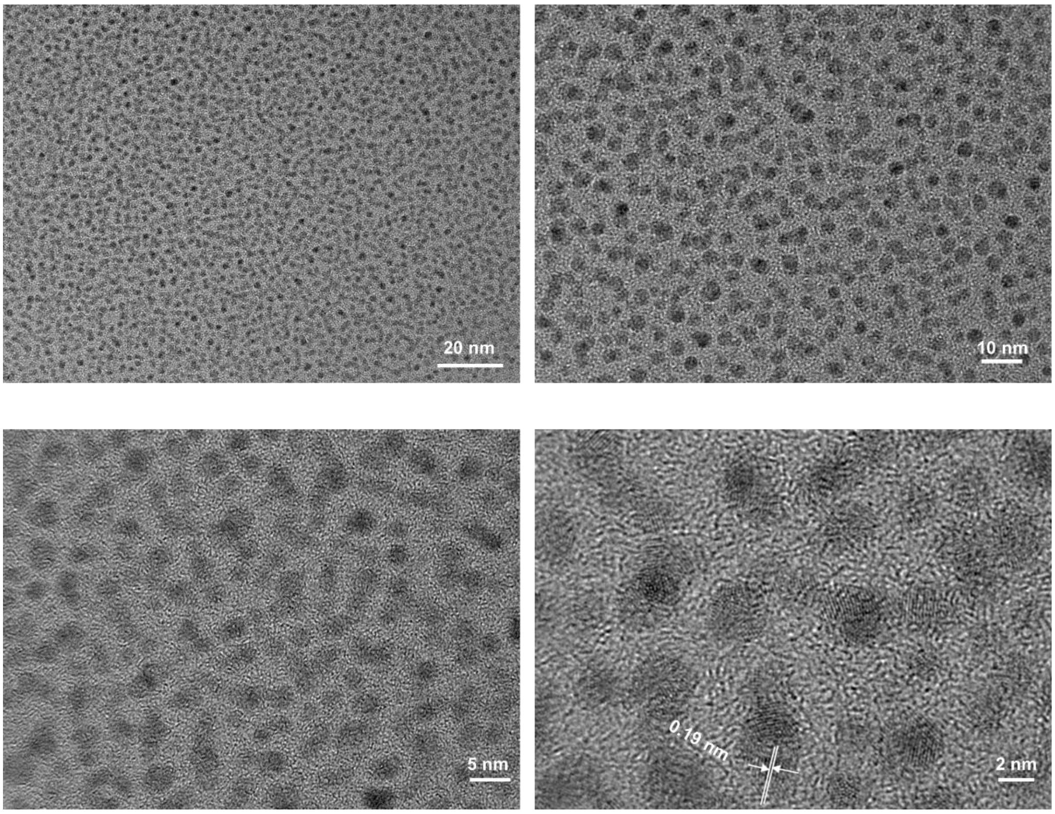

Supplementary Figure 1. The TEM images of CDs.

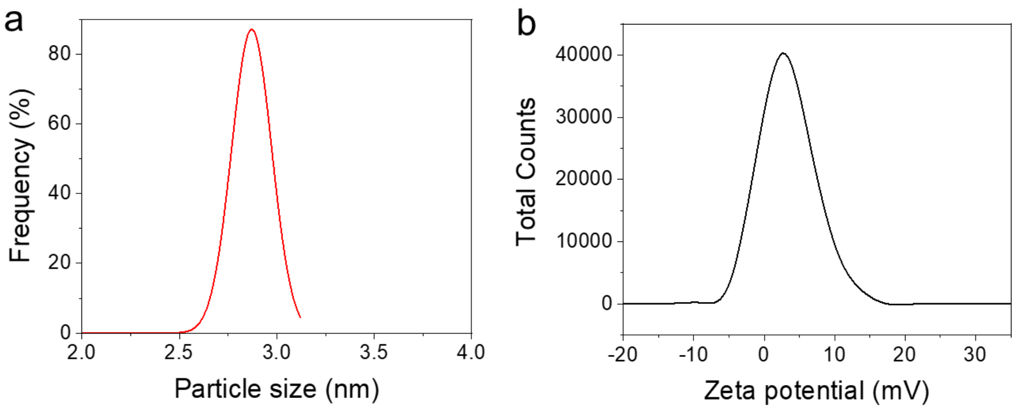

Supplementary Figure 2. (a) Particle size distribution of CDs. (b) Zeta potential of CDs.

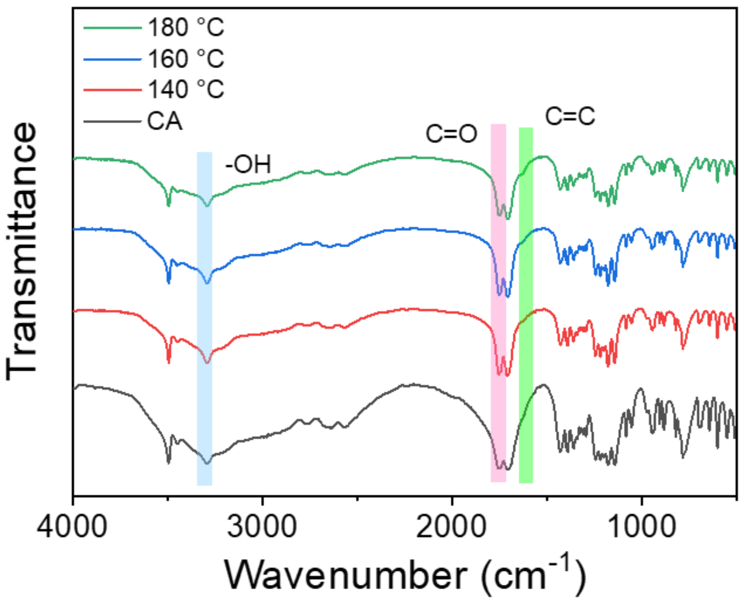

Supplementary Figure 3. FT-IR spectra of CA and CDs prepared at different temperatures.

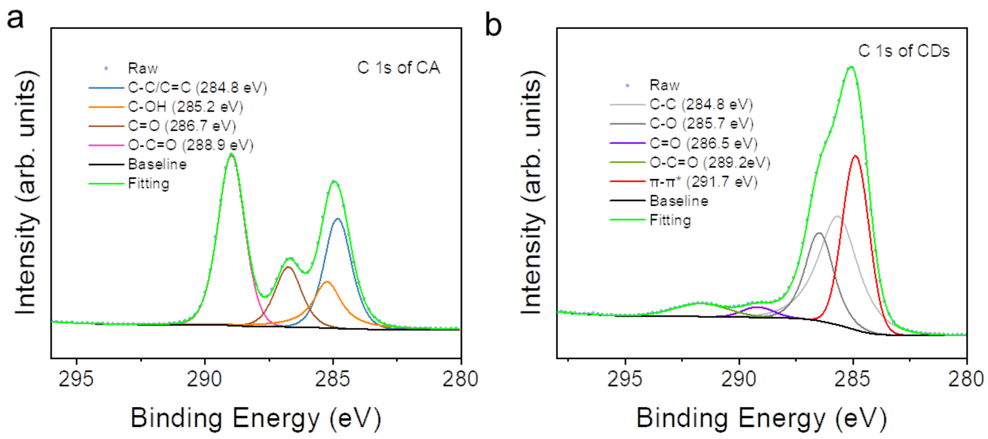

Supplementary Figure 4. XPS analysis of CA and CDs. a C1*s* scan of CA. **b** C1*s* scan of CDs. (C) O1*s* scan of CDs.

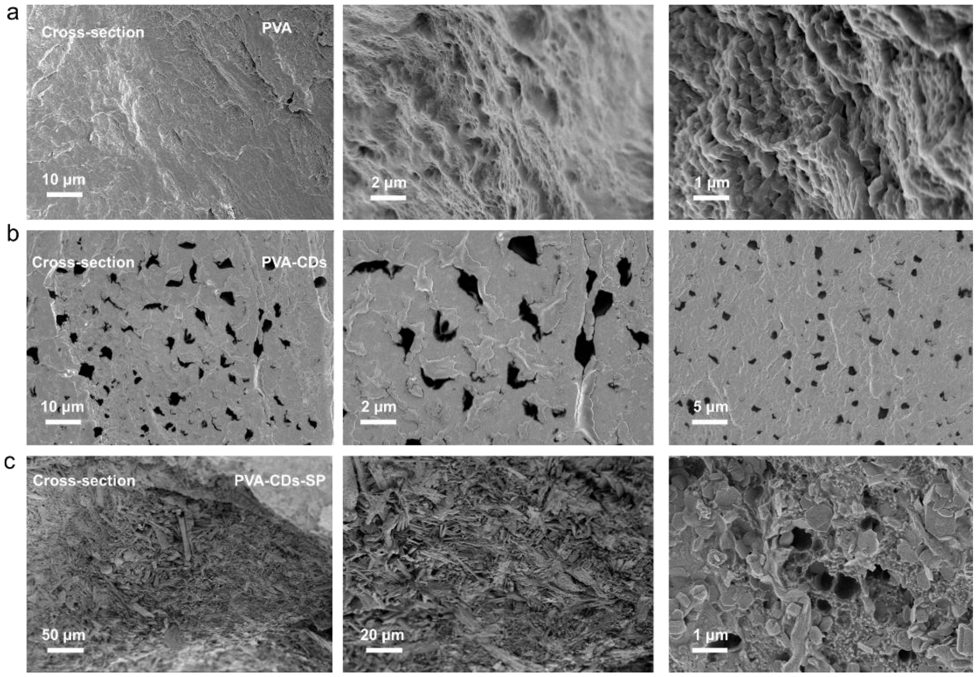

Supplementary Figure 5. SEM of the cross-section of hydrogel. a PVA. **B** PVA-CDs. **c** PVA-CDs-SP.

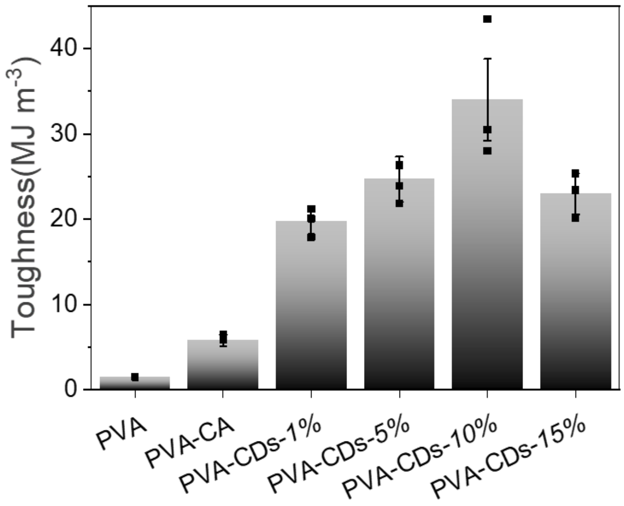

Supplementary Figure 6. Toughness and Young's modulus of PVA, PVA-CA and PVA-CDs at different concentrations of CDs. The bars represent the average values of three independent measurements, with error bars indicating the standard deviation.

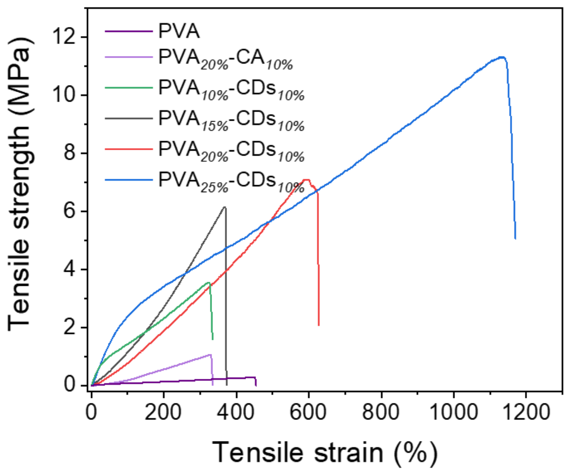

Supplementary Figure 7. Stress-strain curves of polyvinyl alcohol (PVA), PVA-citric acid (PVA-CA), and PVA-carbon dots (PVA-CDs) at different concentrations of PVA.

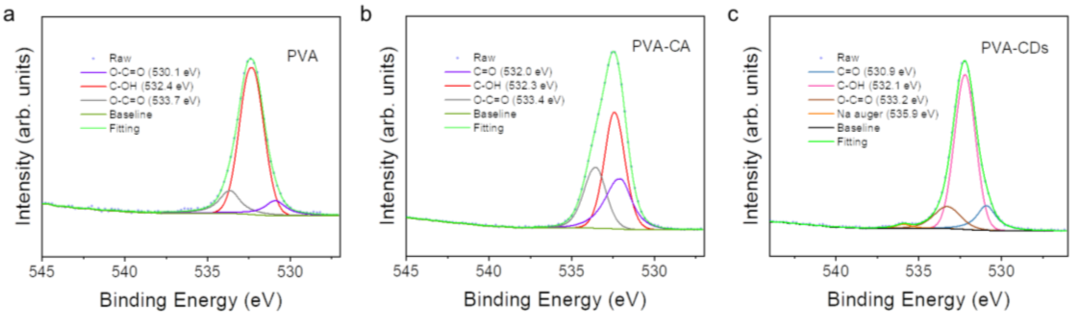

Supplementary Figure 8. XPS analysis of O1*s* scan. a PVA, **b** PVA-CA and **c** PVA-CDs.

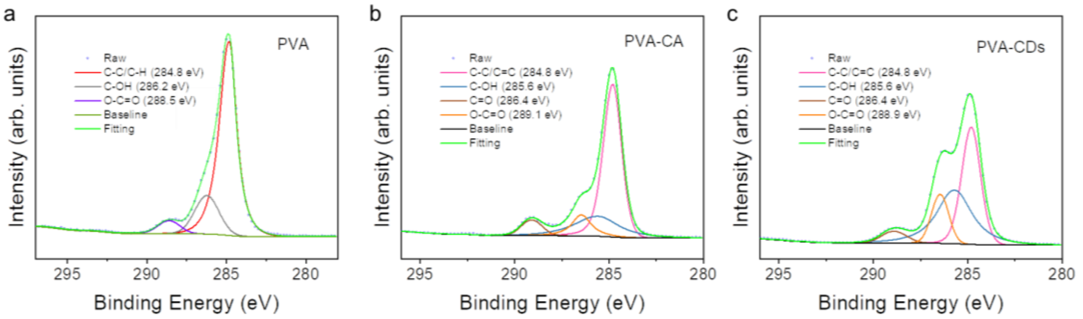

Supplementary Figure 9. XPS analysis of C1*s* scan. a PVA, **b** PVA-CA and **c** PVA-CDs.

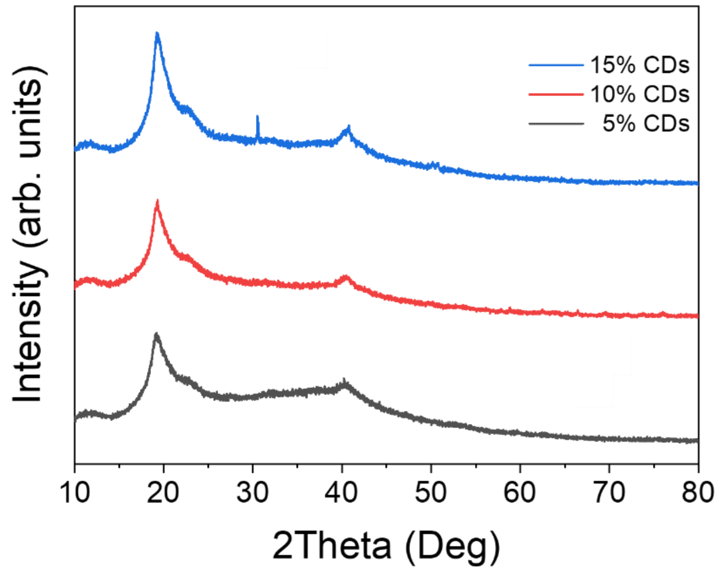

Supplementary Figure 10. XRD spectra of PVA-CDs hydrogels at different content of CDs.

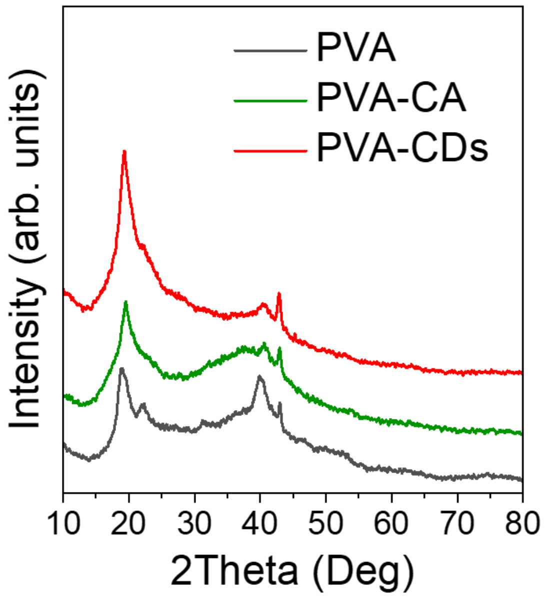

Supplementary Figure 11. XRD profiles of the PVA, PVA-CA and PVA-CDs hydrogel.

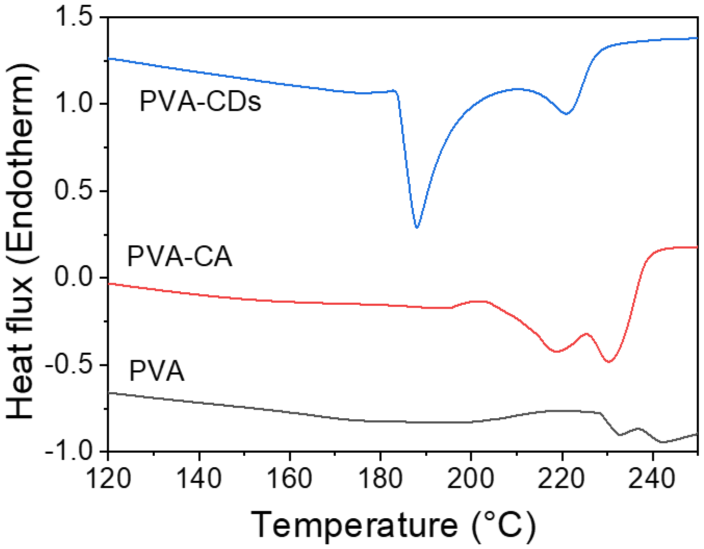

Supplementary Figure 12. DSC curves of PVA, PVA-CA and PVA-CDs hydrogels.

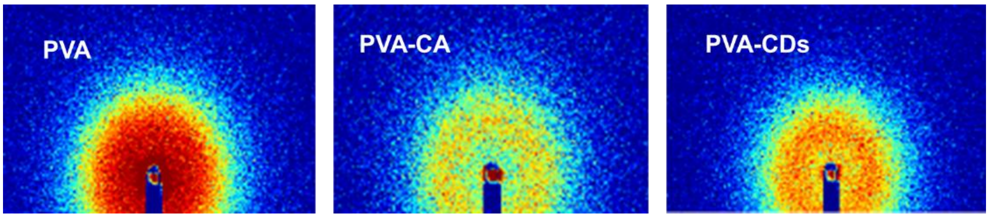

Supplementary Figure 13. 2D-SAXS patterns of the PVA, PVA-CA and PVA-CDs hydrogels.

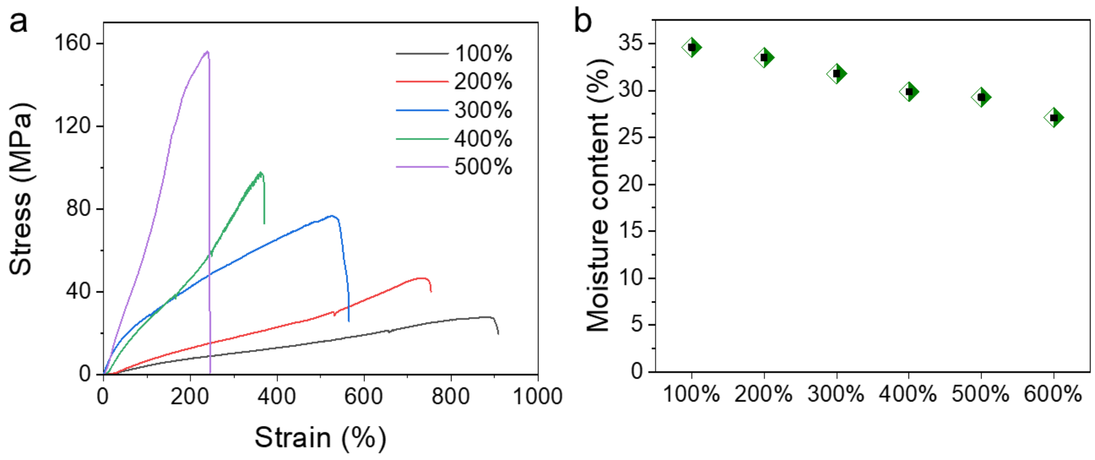

Supplementary Figure 14. (a) Stress−strain curves of PVA-CDs-SP hydrogels at different elongations, and (b) water content of PVA-CDs-SP hydrogels at different elongations.

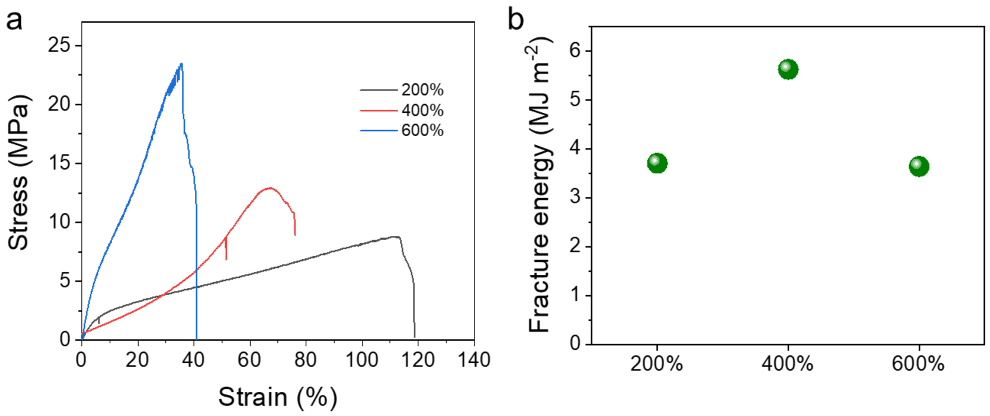

Supplementary Figure 15. (a) Stress–strain curves of notched PVA-CDs-SP hydrogels at different elongations. (b) Fracture energy of notched PVA-CDs-SP hydrogels at different elongations.

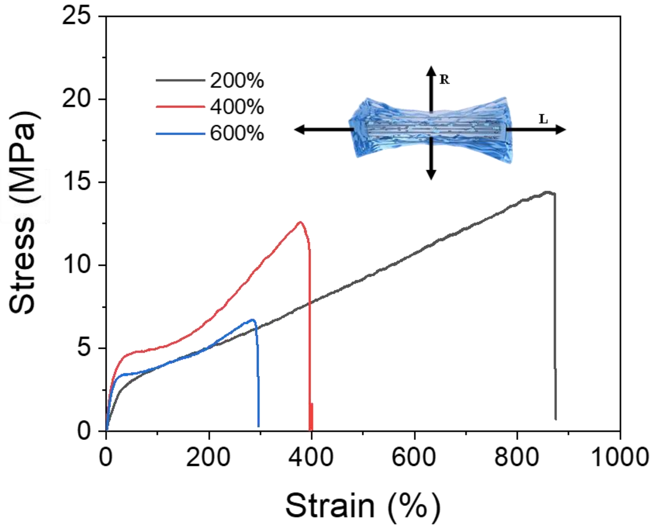

Supplementary Figure 16. Stress−strain curves of PVA-CDs-SP hydrogels in the vertical direction (R direction) at different elongations.

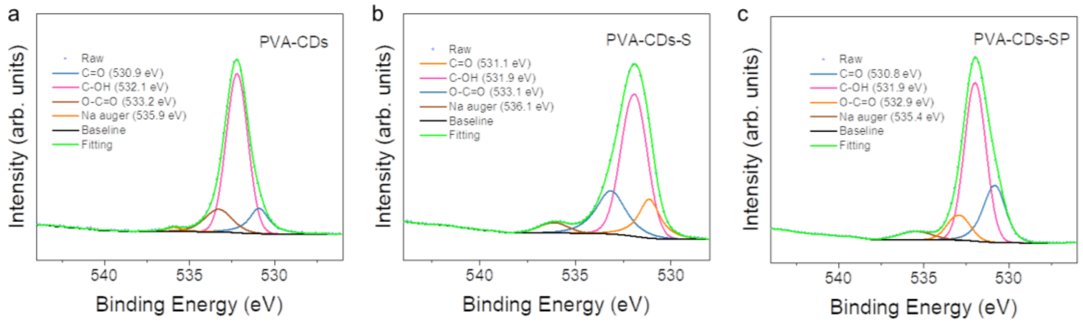

Supplementary Figure 17. XPS analysis of O1*s* scan. a PVA-CDs, **b** PVA-CDs-S and **c** PVA-CDs-SP hydrogels.

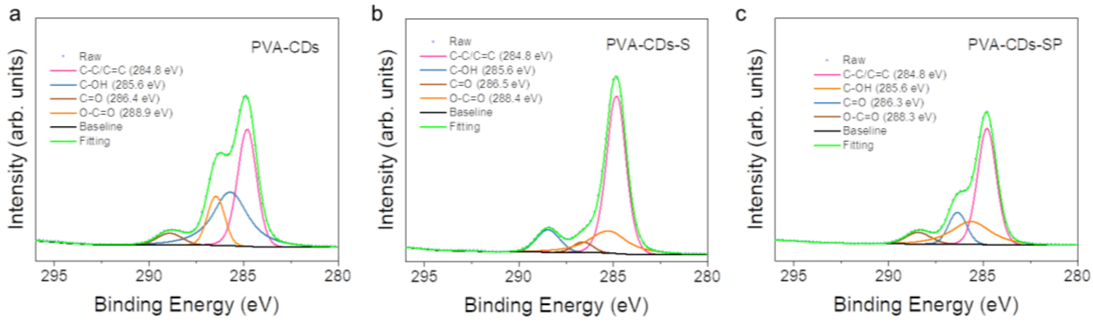

Supplementary Figure 18. XPS analysis of C1*s* scan. a PVA-CDs, **b** PVA-CDs-S and **c** PVA-CDs-SP hydrogels.

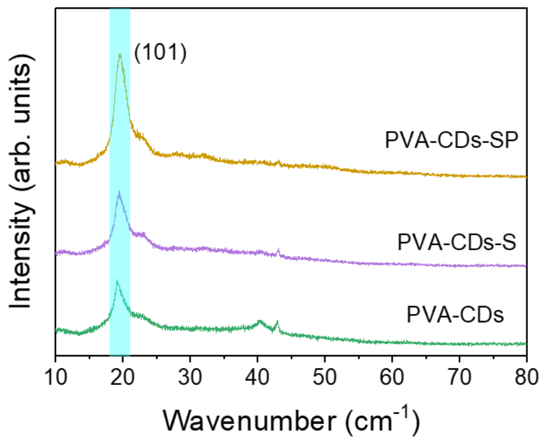

Supplementary Figure 19. XRD profiles of the PVA-CDs, PVA-CDs-S and PVA-CDs-SP hydrogels.

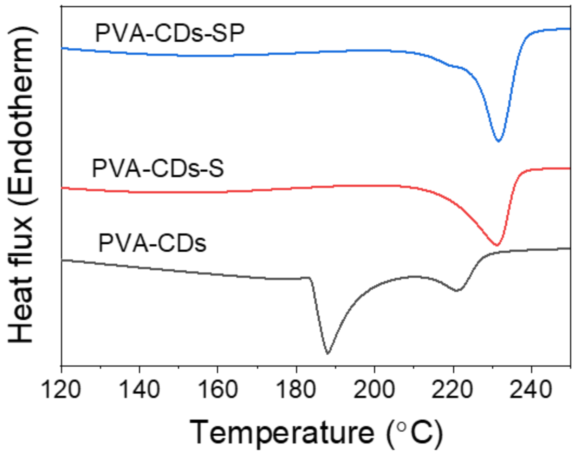

Supplementary Figure 20. DSC curves of PVA-CDs, PVA-CDs-S and PVA-CDs-SP hydrogels.

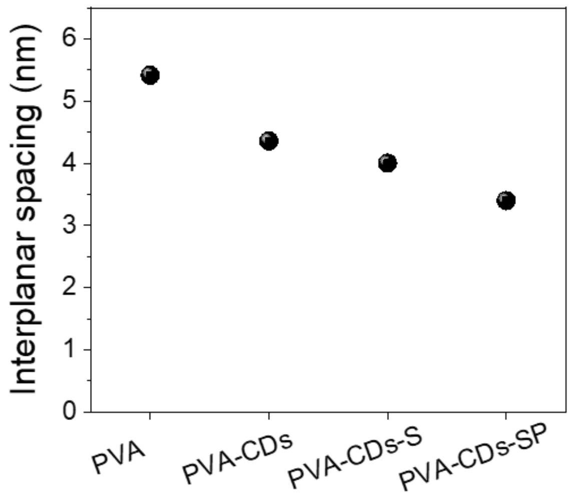

Supplementary Figure 21. The average distance between neighboring crystalline domains in the PVA, PVA-CDs, PVA-CDs-S and PVA-CDs-SP hydrogels.

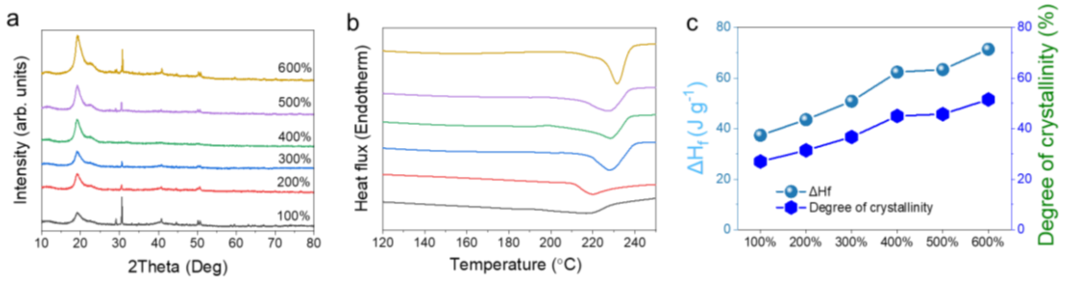

Supplementary Figure 22. (a) XRD spectra of PVA-CDs-SP hydrogels at different elongations. (b) DSC curves of PVA-CDs-SP hydrogels at different elongations. (c) Enthalpy change and crystallinity of PVA-CDs-SP hydrogels at different elongations.

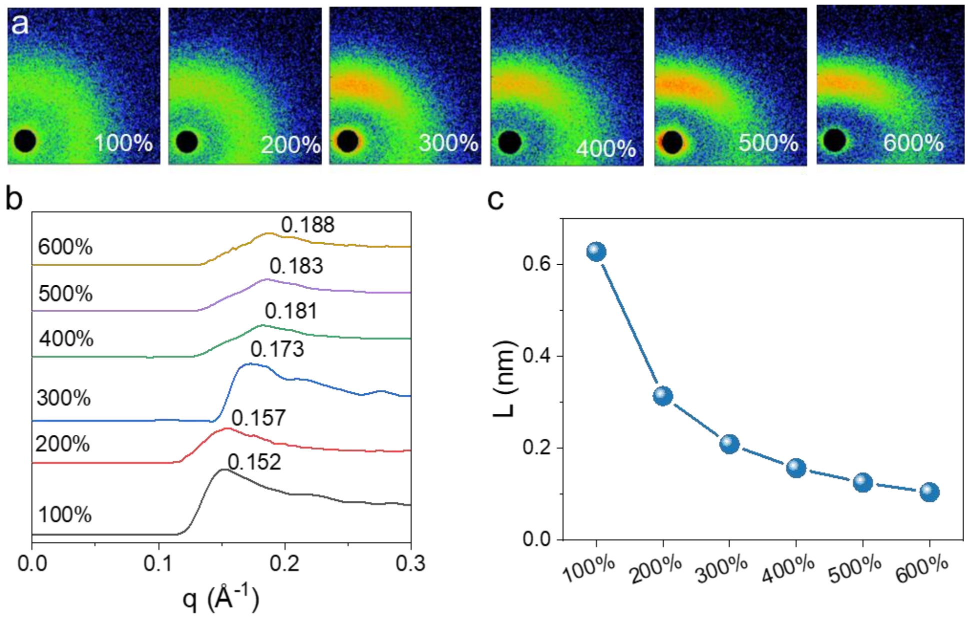

Supplementary Figure 23. (a) 2D-SAXS spectra of PVA-CDs-SP hydrogels at different elongations. (b) SAXS spectra of PVA-CDs-SP hydrogels at different elongations. (c) Interplanar spacing of PVA-CDs-SP hydrogels at different elongations.

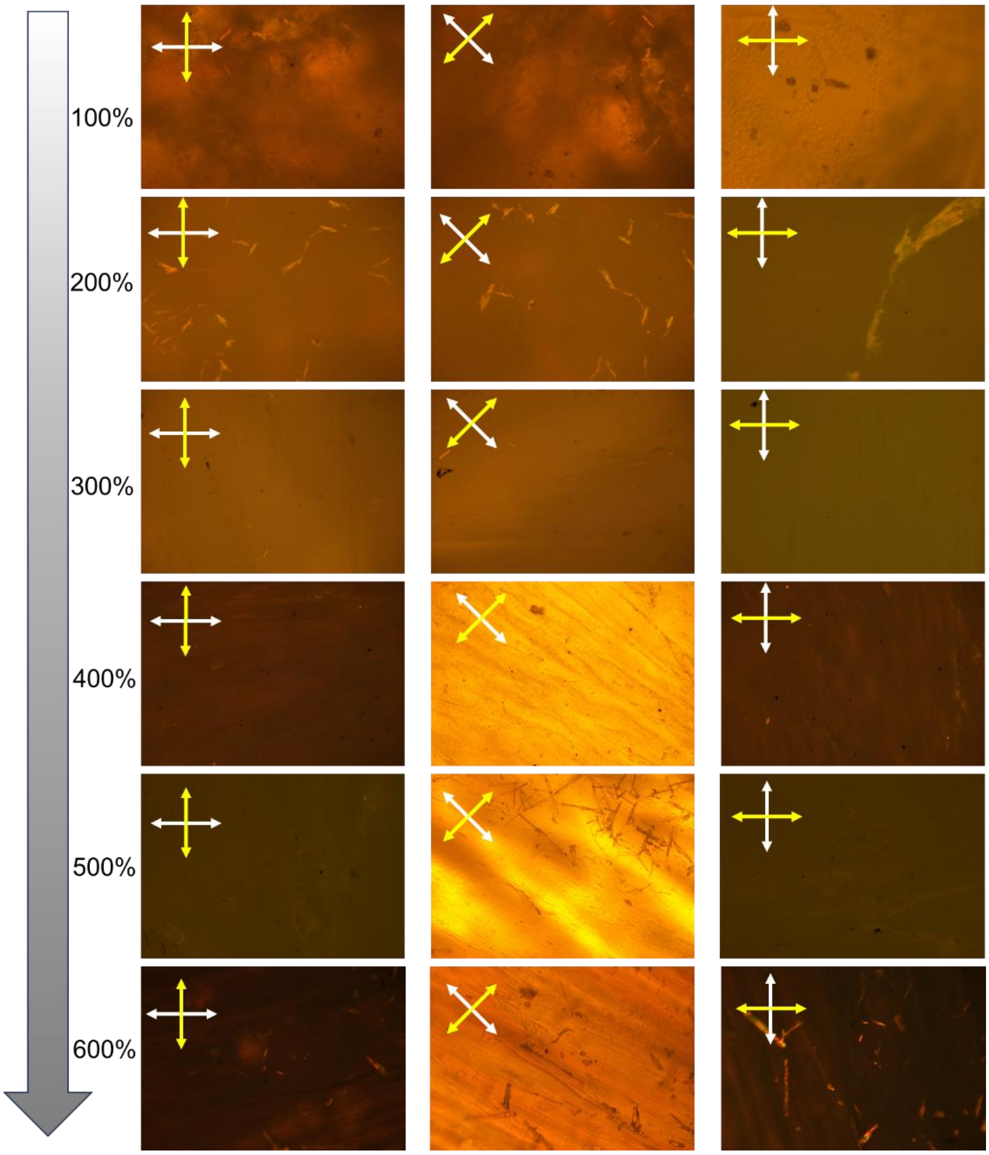

Supplementary Figure 24. Morphologies observed by orthogonal polarizing microscope. For the left sample, randomly placed the sample on the stage and define this position as the 0° reference. Then, rotated the platform to 45° (middle) and 90° (right) sequentially for observation.

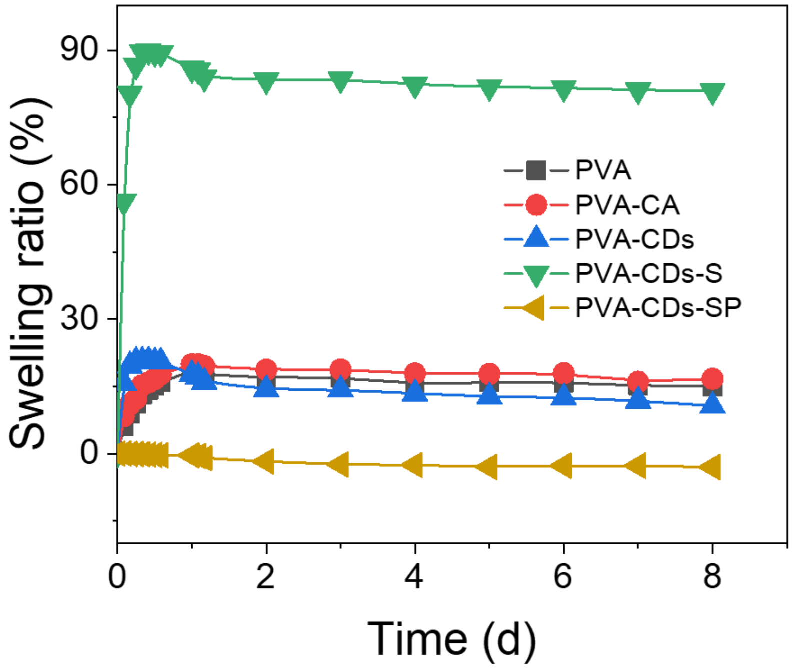

Supplementary Figure 25. Swelling kinetics of PVA, PVA-CA, PVA-CDs, PVA-CDs-S and PVA-CDs-SP hydrogels.

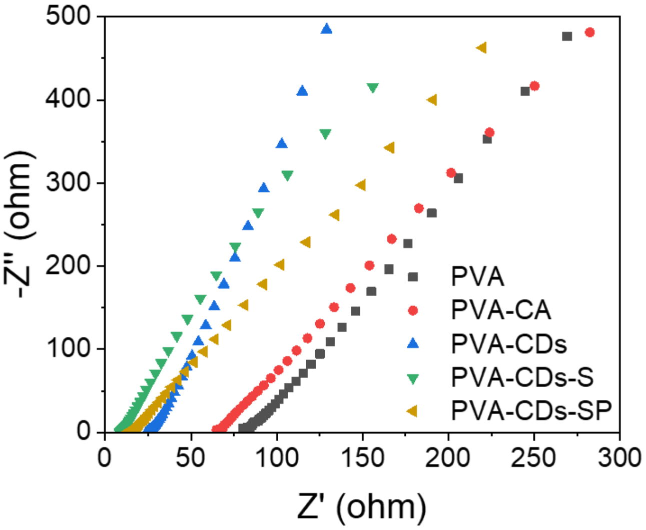

Supplementary Figure 26. Nyquist diagrams at PVA, PVA-CA, PVA-CDs, PVA-CDs-S and PVA-CDs-SP hydrogels.

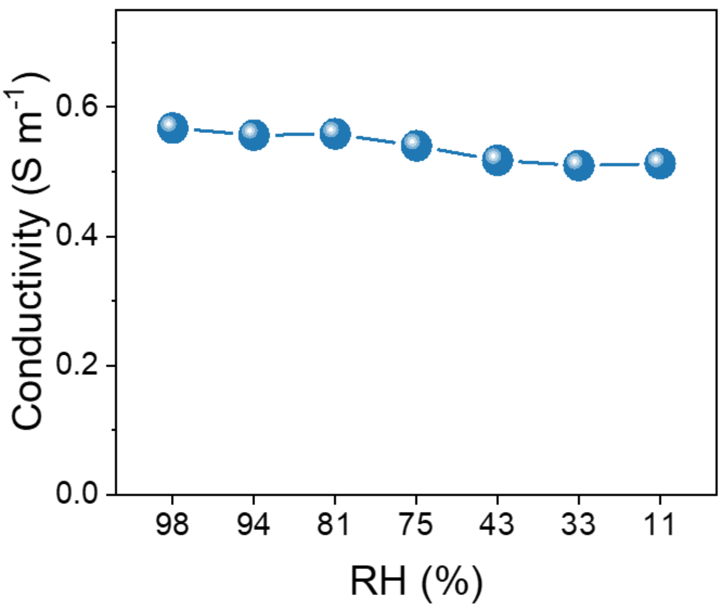

Supplementary Figure 27. Conductivity of PVA-CDs-SP hydrogels at various humidity levels.

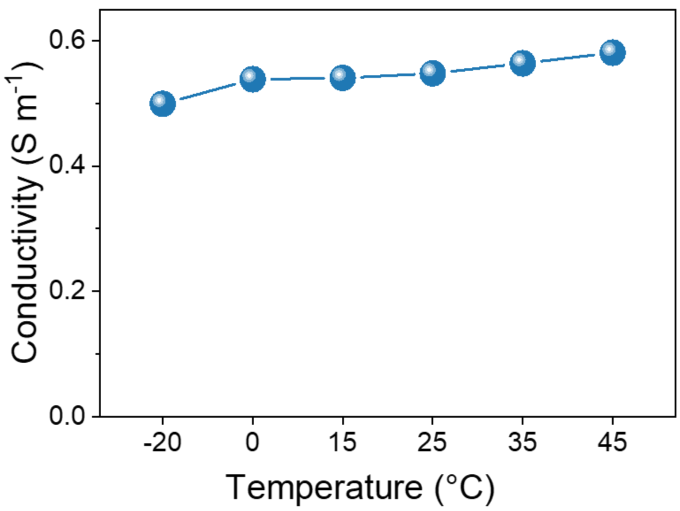

Supplementary Figure 28. Conductivity of PVA-CDs-SP hydrogels at varying temperatures.

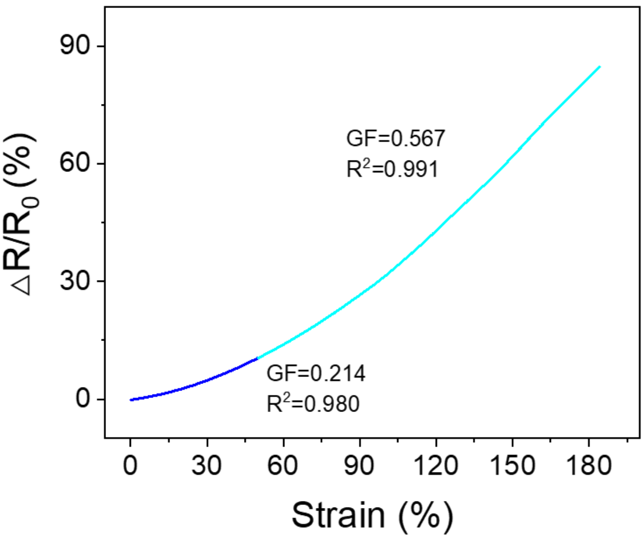

Supplementary Figure 29. Gauge factor of the PVA-CDs-SP hydrogel within the 970% strain range.
