# Supplementary material for: A tough and robust hydrogel constructed through carbon dots induced crystallization domains integrated orientation regulation
Source: Nat Commun. 2025 Jul 5;16:6221. doi: 10.1038/s41467-025-61535-1 (PMC12228686; doi:10.1038/s41467-025-61535-1)
Supplement: Supplementary file 2 — Description of Additional Supplementary Files [file 41467_2025_61535_MOESM2_ESM.pdf]

Description of Additional Supplementary Files

Supplementary Movie 1: The hydrogel bundle can support the weight of an adult male.

Supplementary Movie 2: The hydrogel bundle is capable of pulling a car.
